# Establishing Consensus of Best Practice for CEA Use in Treatment of Severe Burns: A US Burn Provider Delphi Study

**DOI:** 10.1093/jbcr/irae050

**Published:** 2024-03-19

**Authors:** Paul Glat, Lisa Quirk, Scott Hultman, Jennifer Kesey, Arpana Jain, John Griswald, Fitzgerald Natalie, Lucy Wibbenmeyer, Hamed Amani, Caryn Cramer, William L Hickerson

**Affiliations:** Drexel University School of Medicine, Philadelphia, PA, USA; Drexel University School of Medicine, Philadelphia, PA, USA; Duke University School of Medicine, Durham, NC, USA; Texas Tech University Health Science Center, Lubbock, TX, USA; Arizona Burn Center, Pheonix, AZ, USA; Texas Tech University Health Science Center, Lubbock, TX, USA; Richard M Fairbanks Burn Center, Indianapolis, IN, USA; University of Iowa Health Care, Iowa City, IA, USA; Lehigh Valley Health Network Regional Burn Center, Allentown, PA, USA; Vericel Corporation, Cambridge, MA, USA; University of Tennessee Health Science Center (retired), Memphis, TN, USA

**Keywords:** cultured epidermal autograft, severe burns, Delphi, consensus

## Abstract

The goal of this study was to inform standards of best practice in the use of cultured epidermal autograft (CEA), manufactured in the United States, for the treatment of patients with severe burns. The study was designed using the modified Delphi technique, a method for structuring group communication among experts to promote the development of consensus-based recommendations. Known areas of variability related to the stages of CEA treatment were identified by literature review prior to the study and were confirmed through qualitative interview with the experts. The areas included Preoperative Planning/Surgical Planning, Immediate Postoperative Care, and Rehabilitation and Long-Term Care. A list of 22 questions was developed based on interviews with the experts, and a 3-round Delphi technique was used to establish consensus (≥80% agreement). Following 3 rounds (quantitative, qualitative, and virtual roundtable meeting) of the Delphi study, important guidance for the use of CEA treatment in severely burned patients gained consensus. Final key recommendations included minimum burn limit for CEA treatment (30%-50% TBSA), ideal biopsy timing (1-2 days), number of grafts (enough to cover; adjust 72 hours before application), use of dermal substrates (recommended) and wide meshed autograft underlay (recommended), optimal CEA drying time per day (open air >6 hours), slings used if CEA placed on extremities (recommended), dressing changes (performed every day, all at once, with all layers removed down to bridal veil), CEA backing removal (10-14 days after placement), heat lamps (can be used to aid the wound in drying, depending on clinical judgment), initial activity restrictions lifted (beginning 10 days after backing removal), compression garments (introduced at approximately 2 months post-CEA surgery), and lasers (CO_2_ laser can be introduced between 3 and 6 months post-CEA surgery).

## INTRODUCTION

Cultured epidermal autograft (CEA) is an autologous product manufactured and marketed under the tradename Epicel in the United States by Vericel Corporation (Cambridge, Massachusetts). Cultured epidermal autograft is an aseptically processed skin graft grown in the laboratory, consisting of rectangular sheets of autologous keratinocytes used for permanent burn wound coverage in severely burned patients.^1^ Cultured epidermal autograft is indicated for use in adult and pediatric patients who have deep dermal or full thickness burns comprising a total body surface area (TBSA) ≥ 30%.^2^ While there are multiple publications of larger studies^3–6^ describing the detailed use of CEA, surgeons and nurses in real-world practice settings report utilizing CEA differently across burn centers in terms of preoperative planning, patient selection, dermal substrates, frequency of use, and patient rehabilitation. Feedback from key burn centers and trauma surgeons indicated that an expert panel in the format of a Delphi Study would provide important evidence to support potential practice norms in treatment and rehabilitation in the use of CEA. The goal of the study was to inform standards of best practices for CEA treatment of severe burns in the United States via a multiphased consensus approach using CEA experts.

## METHODS

### Study design

In our study, we used the modified Delphi technique,^7^ a method for structuring group communication among experts to promote the development of consensus-based recommendations. The Delphi survey method is used for collecting opinions from experts by using a number of questionnaire rounds, feedback on responses, and the opportunity for participants to modify their responses.^7^ This method uses a systematic progression of repeated rounds of voting and is an effective process for determining expert group consensus where there is limited definitive evidence, defining consensus among the expert panel as 70%-80%.^8–11^ The full evaluation requires various methods, including review of the literature, interviews with experts, and iterative consensus of an expert panel.^8,9^

Burn surgeons with experience performing CEA procedures and knowledge of best practices were invited to participate in three rounds of consensus-building. The 3 rounds were planned to obtain feedback from the experts, including 1 qualitative 60-minute virtual interview to identify current CEA use patterns, a 20-minute online quantitative survey, and a virtual round table to align on discrepancies of CEA treatment and rehabilitation practices. Vericel Corporation provided support for the project through the use of an independent consulting company (Trinity Life Sciences, Cambridge, MA) to conduct interviews and moderate the consensus rounds. All 3 rounds were facilitated by Trinity Life Sciences, Cambridge, Massachusetts. Trinity Life Sciences is a global team of advisors, technical experts, and data specialists who provide evidence-based solutions to life science companies. Following completion of each round, data were analyzed and summarized by Trinity Life Sciences. For the virtual round table (round 3), consensus was obtained by the moderator through verbal acceptance/lack of acceptance (yes or no) at the end of the discussion of each subpart. The moderator ensured at least 5 of the 6 attending surgeon panel members (~83%) agreed with the recommendations before moving to a discussion of the next consensus topic.

Four known areas of variability (domains) related to the stages of CEA treatment were identified by literature review prior to the study and were confirmed in round 1 through qualitative interview (Table 1). The domains included Preoperative planning/surgical planning, Immediate postoperative care, and Rehabilitation and long-term care. Within each of the domains were subtopics that may be dependent on patient characteristics. Subtopics of interest for our study were chosen via a selection process with the Vericel team, in which 26 key topics were narrowed down and prioritized to the top 13. These subtopics include Minimum %TBSA, Time to Biopsy, Dermal Substrate, Timing to Graft Planning, Additional Number of Grafts, Underlay, Daily Graft Drying Time, Dressing Strategy, Timing for Backing Removal, Use of Bili lights/Drying lamps, Techniques for Graft Shearing and Blistering, Activity Restrictions, and Compression Garments and Laser Use.

**Table 1.** Domains and Subtopics for CEA Consensus Guidelines

Domains		
Preoperative planning/surgical planning	Immediate postoperative care	Rehabilitation and long-term care
**Subtopics**		
Patient selection: % TBSA to treat a patient with CEA	Daily graft drying time	Activity restrictions
Ideal timing of biopsy	Postoperative dressing strategy	Compression garments and laser use
Choice of dermal substrate	Timing of backing removal	
CEA graft planning	Use of bili lights and heat lamps during Week 1 Use of lights after takedown of backing	
Use of underlay with CEA	Techniques to avoid graft shearing and blistering	

### Expert panel

There are no defined rules for the size of an expert panel. Linstone^9^ et al. suggest a minimum of 7 experts. The community of burn surgeons and nurses in the United States is small; therefore, it was decided a priori that recruitment would be conducted between July 2022 and November 2022 with a goal of recruiting a minimum of 7 participants (surgeons and nurses) who would be likely to continue through all 3 rounds of the Delphi study.

Vericel has a record of the number of patients treated at each burn center per year due to the autologous nature of the product. Surgeons and nurses invited to participate were experienced CEA users recruited by Trinity Life Sciences using a contact list compiled by Vericel. Key criteria for inclusion in the study included: working at an American Burn Association (ABA) verified burn center, working for a minimum of 3 years postresidency, and having treated a minimum of 2 patients with CEA over the past 2 years.

A total of 14 experts (11 surgeons and 3 nurses), proficient in CEA use, agreed to participate in the study. The surgeons had an average of 14 years in practice. Among the 11 surgeons, a total of 289 patients had been treated in the past year with TBSA >30% and 160 patients with TBSA >40%. A total of 53 patients had been treated with CEA by the surgeons in the past 2 years. Experts were from across the United States including the South, Mid-Atlantic, and Southeast (21.3%, each geographical location), Pacific Northwest (14.3%), and Midwest, Southwest, and Northeast (7.1%, each). Of the 14 experts recruited, 11 participated in rounds 1 and 2, and 8 (6 surgeons, 2 nurses) participated in round 3. Nurses and surgeons answered the same questions in the 2-round interview stage, and both contributed to the virtual discussion in round 3.

## RESULTS

### Round 1

A 60-minute virtual interview followed a discussion of the overall process of CEA use, from preoperative planning to patient discharge. The interview was conducted by a trained moderator from Trinity Life Sciences. Surgeons and nurses were asked to provide detailed responses on CEA best practice methods during preoperative planning, surgical planning, postoperative care, and rehabilitation and long-term care. Responses and information obtained during the interviews in Round 1 were used to compose the online survey for round 2.

### Round 2

The 20-minute online survey was fielded from October 3, 2022 to November 1, 2022. Patient profiles, designed to capture the array of potential patient situations that surgeons may encounter when utilizing CEA in the real-world setting, were used to stimulate a guided discussion. All surgeons and nurses from Round 1 were emailed a link to participate, and 11 (9 surgeons, 2 nurses) participated. Percentages are based on the 9 surgeon online respondents.

The experts answered a multi-select response survey based on expert responses from Round 1. A total of 19 questions (16 to establish guidelines and 3 to gain additional information) were asked during Round 2. The questions and expert responses are shown in Table 2.

**Table 2.** Consensus Questions for Round 2

Question^a^	Panelist response	Consensus reached?
1. *What is the absolute minimum TBSA% burn at which you consider treating a patient with Epicel?*		No
a. *30%–40%*	a. 11%	
b. *41%–50%*	b. 22%	
c. *51%–60%*	c. 33%	
d. *61%–70%*	d. 22%	
e. *>70%*	e. 11%	
2. *How soon after admission do you typically procure a biopsy for Epicel?*		No
a. *1–2 days*	a. 56%	
b. *3–4 days*	b. 22%	
c. *5–7 days*	c. 11%	
d. *>7 days*	d. 11%	
3. *Do you use a dermal substrate for wound coverage before using Epicel?*		Yes
a. *Yes*	a. 78%	
b. *No*	b. 22%	
4. *Which dermal substrate do you use prior to Epicel placement?*		No
a. *Allograft*	a. 67%	
b. *BTM (biodegradable temporizing matrix)*	b. 56%	
c. *Integra*	c. 44%	
*[Note: experts made more than once choice for this question]*		
5. *How long after patient admission do you decide the number of Epicel grafts needed?*		No
a. *Within 1 week*	a. 44%	
b. *Between 1 and 2 weeks*	b. 22%	
c. *After 2 weeks*	c. 33%	
6. *What percentage of additional graft area do you typically order?*		No
a. *<5%*	a. 11%	
b. *5%–10%*	b. 33%	
c. *>10%*	c. 33%	
d. *Do not order extra*	d. 22%	
*Note: 77% of respondents order additional grafts but only 33% order between 5% and 10% as recommended.*		
*7. Informational question only regarding number of grafts used in a surgery*	N/A	
	Informational only	
8. *Do you use a skin graft base/underlay when applying Epicel?*		Yes
a. *Yes*	e. 100%	
b. *No*	f. 0	
9. *What types of underlays do you most frequently use for Epicel?*		No
a. *RECELL*	a. 11%	
b. *Split thickness skin graft (4:1)*	b. 33%	
c. *Split thickness skin graft (6:1)*	c. 67%	
d. *MEEK*	d. 0	
10. *How long do you leave Epicel areas open to air?*		Yes
a. *<4 hours/ day*	a 0.0	
b. *4–5 hours/ day*	b. 11%	
c. *6–8 hours/ day*	c. 78%	
d. *>8 hours/ day*	d. 11%	
11. *Do you use slings for drying Epicel grafts after placement located in the extremities?*		Yes
a. *Yes*	a 0.89%	
b. *No*	b. 11%	
12. *When do you perform dressing changes?*		Yes
a. *Every day*	a 100%	
b. *Every other day*	b. 0	
13. *How do you perform dressing changes?*		Yes
a. *All at once*	a 89%	
b. *In stages*	b. 11%	
14. *When you do a dressing change postoperation, do you remove all layers down to the bridal veil?*		Yes
*a. Yes*	a 100%	
*b. No*	b. 0	
15. *What solutions do you use in your immediate postoperative dressings?*		
a. *Silver nitrate*	N/A	
b. *Sulfamylon solution*	Informational only	
c. *Vashe product*	Highest response:	
d. *Other antifungal antibiotic solution*	Sulfamylon solution (56%)	
e. *No ointment used*		
*[Note: experts made more than once choice for this question]*		
16. *How long do you wait for backing removal?*		No
f*. <7 days*	a 11%	
g. *7–10 days*	b. 67%	
h. *11–14 days*	c. 0	
i. *>14 days*	d. 22%	
17. *Do you use “bili” lights for Epicel?*		No
a. *Yes*	a 44%	
b. *No*	b. 56%	
18. *Do you use drying lamps for Epicel?*		No
a. *Yes*	a 56%	
b. *No*	b. 44%	
19. *What dressings/ointments do you use for graft sheering and blistering?*		
a. *Aquaphor*		
b. *Vaseline*	N/A	
c. *Bacitracin*	Informational only	
d. *Xeroform*	Highest response:	
e. *Polysporin*	bacitracin (56%)	
f. *Adaptic*		
g. *Coconut oil-based lubricant*		
20. *When do you typically start to remove activity restrictions?*		No
a. *<7 days*	a 22%	
b. *7–10 days*	b. 33%	
c. *11–14 days*	c. 22%	
d. *>14 days*	d. 11%	
21. *At what point do you typically introduce compression garments for Epicel patients?*		No
a. *<2 months*	a 56%	
b. *2–3 months*	b. 44%	
c. *3–6 months*	c. 0	
d. *>6 months*	d. 0	
22. *At what point do you typically introduce laser treatment for Epicel patients?*		No
a. *<3 months*	a 22%	
b. *3–6 months*	b. 44%	
c. *>6 months*	c. 33%	

^a^Most questions had an option for “other (please specify).”

### Round 3 (Virtual roundtable)

The 2-hour virtual roundtable was conducted on November 14, 2022 led by an expert medical moderator from Trinity Life Sciences. Case presentations were made by all 6 surgeons attending the virtual round table prior to taking the survey in round 3. The topics that were discussed were those that had not achieved consensus via the quantitative survey and additional topics from round 2 that the Vericel team believed could be useful informationally.

**Consensus topics**: Minimum % TBSA, Time to Biopsy, Timing to Graft Planning, Number of Additional Grafts, Types of Dermal Substrates, Types of CEA Underlay, Graft Drying Time, Timing for Backing Removal, Use of Bili lights/Drying lamps, Activity Restrictions, Compression Garments and Laser Use.

**Informational topics**: Techniques for Graft Shearing and Blistering.

At the 2-hour panel meeting, consensus guidelines on best practices for CEA treatment, including details for each phase from preoperative planning through immediate postoperative care and return to full activity were agreed. Consensus was reached during rounds 2 and 3 at 100% for all subtopics with the exception of “dressing changes all at once or in stages” and “timing for backing removal”; however, both reached a consensus of ≥ 80%. The details of the final results and recommendations of the consensus guidelines as determined in the round 3 virtual meeting are provided in Table 3. Additional clarifying information received from physicians and recorded during the round 3 discussion is also provided.

**Table 3.** Final Recommendations for CEA Treatment of Severe Burns

Topic	Final recommendations	Panelist agreement after round 3
Minimum %TBSA for CEA use	❖ Minimum TBSA range for CEA use 30%–50% TBSA	100%
Ideal timing of biopsy	❖ Biopsies are taken within 1–2 days of admission, or as soon as CEA is considered.	100%
Optimal timing to determine the number of CEA grafts to order	❖ Number of CEA grafts required for wound coverage should be determined within a week of patient admission.	100%
Use of dermal substrate under CEA	❖ Yes; dermal substrates under CEA are used by all panelists (types are discussed below in the table).	100%
Should skin graft base be placed under CEA	❖ Yes; skin graft base should be placed under CEA.	100%
Optimal CEA graft drying time	❖ CEA areas should be left open to air >6 hours per day.	100%
Sling use for drying CEA grafts	❖ Yes; slings are used after placement of CEA located in the extremities.	100%
Timing of dressing changes?	❖ Dressing changes are performed every day.	100%
Should dressing changes be performed all at once or in stages?	❖ Dressing changes should be performed all at once	89%
When a dressing change is done postoperation, are all layers removed down to the bridal veil?	❖ Yes; all layers are removed down to the bridal veil.	100%
Timing of backing removal?	❖ Backing should be removed between days 10 and 14 (see additional details below in the table)	80%
Use of “bili” lights/drying lamps for CEA?	❖ Yes; either type of lamp may be used to aid the wound in drying.	100%
Activity after backing removal	❖ Initial activity restrictions may be lifted beginning 10 days after backing removal.	100%
Compression garments	❖ Compression garments should be introduced at approximately 2 months post-CEA surgery or when feasible	100%
When can lasers be introduced?	❖ Laser, specifically CO_2_ laser, can be introduced between 3 and 6 months post-CEA surgery	100%

#### Clarifying information from virtual round table

##### PREOPERATIVE AND SURGICAL PLANNING

Physicians indicated that they would treat a typical burn patient with a minimum % TBSA of 30% to 50%; however, pediatric and elderly patients are more likely to be good candidates at the lower end of the range due to having thinner dermis.

Biopsies, to be sent to the Vericel lab for cell expansion, should be taken as soon as possible (within 1 to 2 days of admission) once CEA has been considered for the patient, although surgeons noted that later than this initial period is still not too late. Biopsies taken earlier are more likely to be clean and free of contamination, improving success rates for CEA engraftment. The number of CEA grafts required should be planned within a week of patient admission; the number of CEA grafts can be adjusted up to 72 hours before they are delivered. Surgeons should consider ordering 5% to 10% extra grafts when ordering CEA. This percentage is considered a good “safety net” in case grafts are mishandled or there is more wound area to cover than initially expected. Leftover grafts can be used to cover autograft donor sites.

Typically, surgeons use a combination of allograft, biodegradable temporizing matrix (BTM), and/or Integra, as substrates for CEA. Allograft was identified as the most frequently used, followed by BTM, then Integra.

Skin graft base underlay (STSG) is recommended for use with CEA. One hundred percent (100%) of surgeons indicated they use a skin graft underlay for Epicel (11% RECELL, 33% STSG [4:1], 67% STSG [6:1], and 0 use MEEK). Surgeons use 6:1 split-thickness skin graft (STSG) for patients with a higher TBSA (ie, >50%) due to fewer donor sites, and 4:1 STSG for lower TBSA (30%-50%). There was no specific follow-up on the use of MEEK with CEA since zero (0) surgeons indicated using MEEK as an underlay for CEA,

Techniques that may be used to reduce or prevent graft shearing and blistering were collected as informational data. Bacitracin and Aquaphor are the most commonly used ointments, along with an adaptic dressing designed to conform to the shape of the wound and adapt to the amount of wound drainage produced.

It is crucial to take a swab of the wound bed early in the treatment process to assess and plan for specific bacterial coverage prior to applying CEA. Surgeons recommended taking a quantitative culture postdebridement and pre-CEA placement and sending it for a gram stain test. The test will take ~4-6 hours to receive a predominate organism and will help determine what solution should be used postoperatively. If the culture is not positive, it is better to not treat the patient topically and instead let the cells dry out on their own (pending clinical judgment of CEA graft appearance). Most surgeons set aside a day with the patient for final debridement and wound preparation ahead of planned CEA placement.

## IMMEDIATE POSTOPERATIVE CARE

During round 3, surgeons adjusted the original recommended time frame of “leaving areas open to air >4 hours/day” made in round 2, to a new recommendation of “areas should be left open to air >6 hours/day.” During the discussion, surgeons noted the timing should be adjusted for the surrounding climate, i.e., if the area is humid, some areas of the wound will likely need to be left open longer than 6 hours (frequently between 8 and 10 hours). The use of bili lights or heat lamps during the first week after the takedown of CEA backing can be used to aid the wound in drying. Situations where drying lights may be useful include where the wound is more moist than expected, or for patients who become hypothermic during the drying process. Surgeons should use their own clinical discernment to determine how best to achieve a dry wound bed and can utilize lamps to aid in this as appropriate.

Surgeons indicated Sulfamylon, silver nitrate, and Vashe are the top 3 solutions used for bandaging the wound. Surgeons suggest when using silver nitrate and Sulfamylon, it is important to use the “wring-out” method to remove excess solution from the bandage to ensure that the wound is not too wet. Dressings should be changed every day, and the changes should be done all at once rather than in stages. All layers should be removed down to the bridal veil.

## TIMING OF BACKING REMOVAL

Backing should be removed between days 10 and 14 post-CEA placement unless the wound area is infected or shows cause for concern. In this case, the backing can be removed as early as day 7, or earlier if indicated. Cause for concern would include situations in which the wound is too moist, wet, or showing signs of infection. If this is the case, surgeons recommend removing backing in these areas earlier, and cleaning and treating with topicals.

## REHABILITATION AND LONG-TERM CARE

Initial activity restrictions can start being lifted around 10 days post-CEA placement to reduce the risk of heterotopic ossification, although this will vary significantly depending on the patient. Restrictions are more difficult to lift before 14 days for patients with excisions on the lower extremities, as movement will stress these areas. Younger patients have a greater ability to start movement earlier compared to older patients. Full activity will likely be resumed weeks after the CEA has engrafted, when the patient is able to get off sedation and ventilation.

Compression garments should be introduced around 2 months postsurgery or when feasible. Other compression, such as Tubigrip, can be used on lower extremities and arms prior to 2 months as these are less harsh on the skin.

Laser, specifically CO_2_ laser, can be introduced between 3 and 6 months postsurgery. This will depend on the amount of burn wound scarring. Intense pulsed light laser can be considered around 6 weeks in situations where you want to change the vascularity and reduce scarring. Lasers (and their settings) are not all the same and use should be approached with care.

## DISCUSSION

The primary goal of this consensus study was to establish guidance to support potential practice norms in the United States for the treatment and rehabilitation of serious burns using CEA, by gaining consensus among surgeons and nurses experienced in the use of CEA. Treatment options for larger percentage total body surface area (%TBSA) burns continue to present challenges for treatment, including the limited number of autologous donor sites available for skin grafting, leading to adaptation of earlier innovative treatments, such as STSG,^12^ MEEK,^13,14^ and CEA,^15^ for wound coverage.

To determine levels of consensus in the use of CEA for severe burns, we used a modified Delphi technique for the development of consensus-based guidelines.^8,9^ As noted in Table 2 of the results, consensus was gained on less than half (44%) of the topics in round 2. In round 2, an agreement was generally achieved for topics regarding whether substrates and underlay should be used, the use of slings, and dressing changes. Many of the topics left for discussion in round 3 centered around the CEA (minimum %TBSA for CEA use; timing of CEA biopsy, measurement for number of grafts, and backing removal; use of drying lamps). As in a Delphi study by Gibson et al.,^16^ lack of consensus on some items may have been due to consideration by surgeons of patient factors. The consensus was achieved in most cases in round 3 by widening the range (ie, for %TBSA, timing for backing removal) or simplifying the choice (eg, type of skin underlay) and allowing surgeons to provide additional clarifying information within each domain. At the end of round 3, ≥80% of surgeons agreed with the final recommendations as shown in Table 3. Therefore, we felt the modified Delphi approach worked well in this study to allow consolidation of consensus statements between rounds, allowing surgeons to provide clarification in round 3, and then gaining agreement in the virtual roundtable in round 3.

Published reports of patient cohorts^3–6^ provide important information of detailed surgical methods and outcomes in the use of innovative technologies for the treatment of severely burned patients. In addition, the Instructions for Use provided by sponsors also provide important details in the use of a specific product like Epicel.^2^ The use of a Delphi Study can also add to the sum of knowledge by gaining consensus for the use of the product across experts. Given that the burn community is very small,^9^ and the community of surgeons who use CEA is even smaller, we were fortunate to be able to recruit 11 surgeons and 3 nurses to confirm the areas of variability (domains) associated with CEA treatment, and the resulting subtopics within each domain of preoperative planning/surgical planning, immediate postoperative care, and long-term care. Nine of the 11 surgeons and 2 nurses were able to answer round 2 survey questions, and 6 of the 11 (and 2 nurses) were able to participate in round 3. Important guidance for CEA treatment gained consensus in the 3 rounds including specifics of CEA use, dressings, drying time, compression garments, and timing of return to activity.

### Limitations of the study

Limitations of the Delphi method for gaining consensus guidance include the selection of experts which may create bias, especially in the small population of surgeons available who treat severe burns and use CEA. In addition, 3 of the 9 surgeons from rounds 1 and 2 could not participate in round 3. While the 6 participating surgeons in round 3 reflect the depth of experience in burn care and CEA use of the original group, some surgeons did not have experience with procedures such as use of MEEK with CEA and could not answer specific questions. A recent publication by Craft-Coffman et al. outlining the procedures and timing in the use of MEEK + CEA has been noted by the authors.^17^ Although the modified Delphi method allowed us the flexibility to narrowly modify statements between rounds 2 and 3, the virtual face-to-face consensus meeting became important in establishing consensus. This could have introduced bias in that participants of the meeting may have felt compelled to conform to the group view, which is a criticism of consensus methodology.^18^

## CONCLUSIONS

The primary goal of this consensus study was to establish guidance to support potential practice norms in the United States for the treatment and rehabilitation of patients with serious burns using CEA. The study used the modified Delphi technique, a method for structuring group communication among experts to promote the development of consensus-based recommendations. Following 3 rounds of quantitative (round 1), qualitative (round 2), and virtual roundtable meeting (round 3) with experts, important guidance for CEA treatment gained consensus including specifics of CEA use, dressings, drying time, compression garments, and timing of return to activity.

## Notes

*Author Contributions:* Caryn Cramer (Conceptualization [equal], Methodology [supporting], Writing—original draft [lead]), Paul Glat (Conceptualization [supporting], Investigation [lead], Writing—review & editing [equal]), Lisa Quirk (Conceptualization [equal], Investigation [equal], Writing—review & editing [equal]), Scott Hultman (Investigation [equal], Writing—review & editing [equal]), Jennifer Kesey (Investigation [equal], Writing—review & editing [equal]), Arpana Jain (Investigation [equal], Writing—review & editing [equal]), John Griswold (Investigation [equal], Writing—review & editing [equal]), Fitzgerald Natalie (Investigation [equal], Writing—review & editing [equal]), Lucy Wibbenmeyer (Investigation [equal], Writing—review & editing [equal]), Hamed Amani (Investigation [equal], Writing—review & editing [equal]), and William Hickerson (Investigation [equal], Writing—review & editing [equal])

*Conflict of Interest Statement:* Paul Glat, Lisa Quirk, and Jonh Griswald have no financial disclosures to make in relation to this project. Scott Hultman, Arpana Jain, and William Hickerson have previously received consulting fees from Vericel. Jennifer Kesey and Natalie Fitzgerald were compensated for their time through a third party for the completion of the survey. Lucy Wibbenmeyer has been a past speaker for Epicel; Hamed Amani is on the advisory board for Epicel. Caryn Cramer is an employee of Vericel Corporation. Vericel Corporation provided support for the project through the use of an independent consulting company (Trinity Life Sciences, Cambridge, MA) to conduct interviews and moderate the consensus rounds.
